# Case Report: Prostate Adenocarcinoma With Mucinous Features of Normal-Level Serum PSA, Atypical Imaging, Biopsy-Negative, and Peculiar Urethrocystoscopic Manifestation

**DOI:** 10.3389/fonc.2020.504381

**Published:** 2020-12-23

**Authors:** Yao Zhang, Hua Shen, Kai Liao, Weili Wu, Jiuming Li, Hongbo Yu, Hongfei Wu, Zengjun Wang

**Affiliations:** ^1^ Department of Urology, The First Affiliated Hospital of Nanjing Medical University, Nanjing, China; ^2^ Department of Urology, BenQ Medical Center, The Affiliated BenQ Hospital of Nanjing Medical University, Nanjing, China

**Keywords:** prostate cancer, mucinous adenocarcinoma, PSA, urethrocystoscopic manifestation, transurethral resection of the prostate, MRI, needle biopsy

## Abstract

**Background:**

Mucinous tumors of the prostate are seen as rare morphological variants of prostate carcinoma. Misdiagnosis and missed diagnosis are frequent clinically, especially when the clinical performance appears atypical. Furthermore, there has not been reported about the urethrocystoscopic performance of mucinous adenocarcinoma growing into the prostatic urethra so far.

**Case Presentation:**

The current case report describes a 48-year old Asian male who was hospitalized because of intermittent gross hematuria for more than two months. The patient was diagnosed as prostatic space occupying lesions and an examination of needle biopsy was conducted on him, which did not indicate a definite malignancy. Transurethral plasma kinetic resection of the prostate (TUPKP) was performed for the patient, but the postoperative pathology revealed prostatic adenocarcinoma with mucinous features. Specifically, two cord-like neoplasms, extending to the bladder neck, were found through urethrocystoscopy in the prostatic urethra, both of which grew pedicles. The pedicles were situated on the right side of the parenchyma of the prostate. Finally, the patient underwent radical prostatectomy three weeks later.

**Conclusion:**

Here, we reported a case that prostatic adenocarcinoma with mucinous features was diagnosed after TUPKP. The patient had normal serum prostate-specific antigen levels with atypical images and negative biopsy result. This report lays stress on the vigilance of clinicians in prostate mucinous adenocarcinoma and makes a description of its peculiar urethrocystoscopic manifestation, typical imaging, and unique growth pattern for the first time.

## Introduction

The primary mucinous tumors of the prostate include mucinous adenocarcinoma of the prostate (MCP), prostatic adenocarcinoma with mucinous features (PCMF), and mucinous adenocarcinoma of the prostatic urethra (MCPU) (1, 2). MCP is extremely rare, with an incidence rate ranging from 0.21–1.10%. Mucinous adenocarcinoma of the prostate is defined as a primary prostatic acinar tumor, characterized by the presence of more than 25% of the tumor composed of glandular tissue with extraluminal mucin. This diagnosis can only be made in radical prostatectomy specimens. Other prostate specimens, including biopsy and transurethral resection, are able to at best confirm the diagnosis of PCMF (3–7). Clinicians and pathologists are often likely to misdiagnose or miss the diagnosis of this disease due to the deficiency in due awareness of its uncommon presentation (8). The results and prognostic significance of it have not been fully understood. Moreover, to the author’s knowledge, urethrocystoscopy of these kinds of adenocarcinoma, which grow into the prostatic urethra, has not been previously reported.

## Case Presentation

A 48-year old male patient from Asia was admitted to the author’s hospital, complaining for more than two months about intermittent gross hematuria accompanied by bulky and dark red clots. The patient also suffered from hemospermia without painful ejaculation during this period and there was no special family or social-related history. A rectal examination suggested a mild enlargement of the prostate, and the central groove was accessible. An irregular and hard mass of about 4 cm in diameter was palpable on the right prostate lobe.

**Figure 1:**
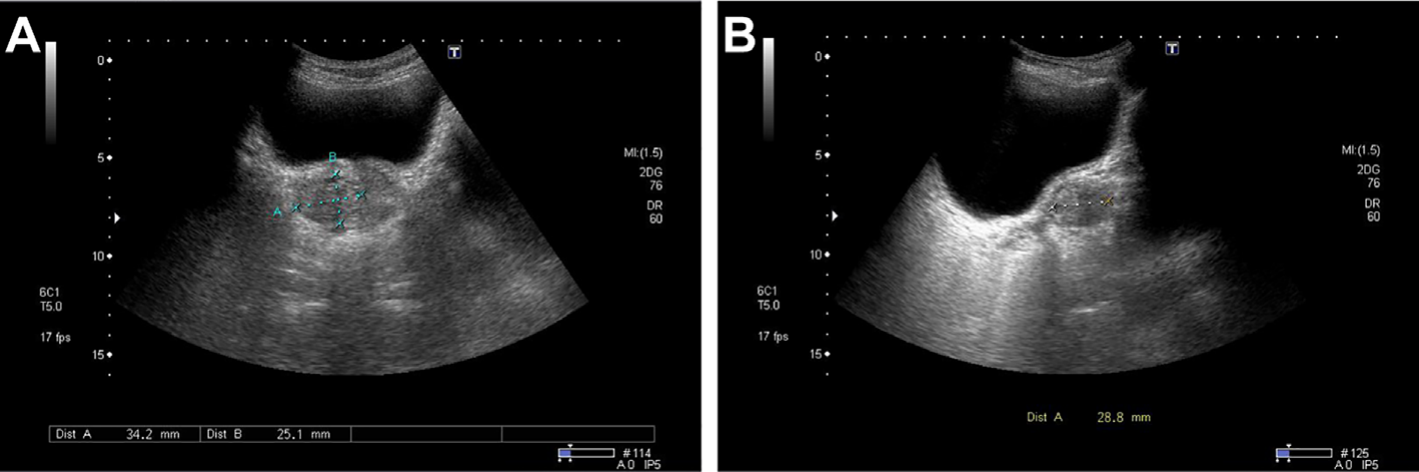
Ultrasound detected a non-uniform hypoechoic nodule, about 34 mm × 25 mm × 29 mm in size, in the right lateral lobe of the prostate with the obscure boundary. No marked color flow signal was observed within the lesion upon Color Doppler flow imaging. **(A)** Transverse position. **(B)** Sagittal position.

**Figure 2:**
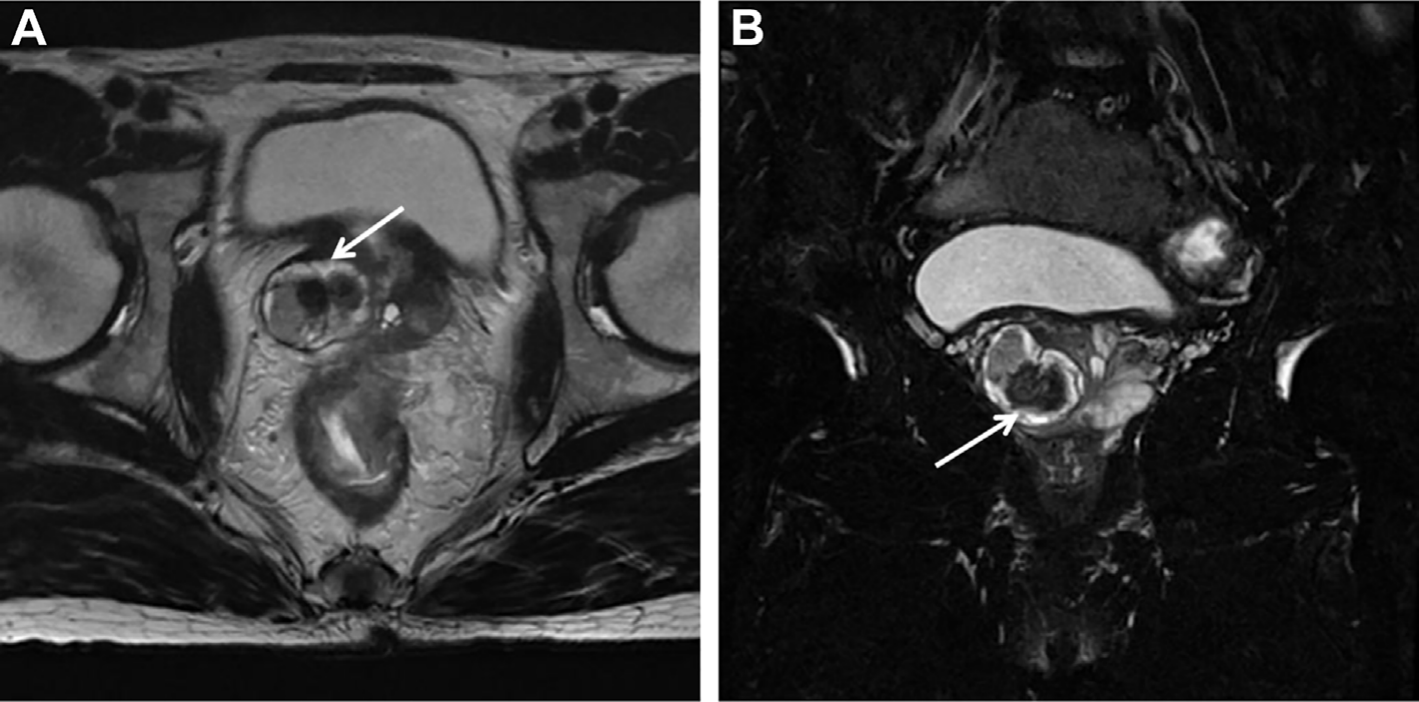
MRI detected a round-like mixed signal lesion in the right lobe of the prostate. T2WI showed mixed signal, with high signal around the periphery and a cluster-like low signal in the center. The lesion boundary was clear, with visible capsule, and the diameter was about 36 mm. The right peripheral zone of the prostate was compressed. **(A)** Axial T2-weighted Image. **(B)** Coronal Fat-suppressed T2-weighted Image.

**Figure 3:**
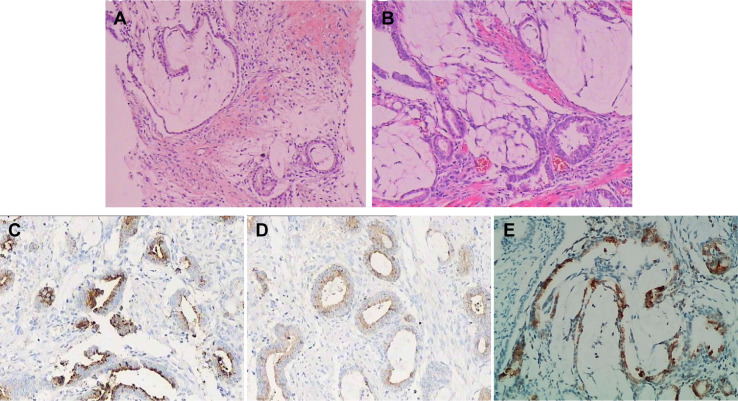
Histopathological and immunohistochemical findings of the tumor. **(A)** Hematoxylin and Eosin stained section of needle biopsy found prostate tissue with interstitial edema around the acinar, part of which showed mucus edema-like changes. **(B)** Hematoxylin and Eosin stained section of TUPKP specimen manifested multifocal mucinous adenocarcinoma with diffuse infiltration. GS was 4 + 3 = 7. Immunohistochemical staining showed positive for PSA **(C)** and PSAP **(D)**, and MUC2 staining showed ∼20% positivity **(E)**.

Ultrasonographic examination indicated benign prostatic hyperplasia and a prostatic space occupying lesion (**Figure 1**). Magnetic resonance imaging (MRI) manifested a prostatic space occupying lesion, presenting mixed signals, with a strong signal around the periphery and cluster-like low signals in the right lobe, at a diameter of about 36 mm (**Figure 2**). The total value of prostate-specific antigen (tPSA) was 2.28 ng/mL, the value of free prostate-specific antigen (fPSA) was 0.267 ng/mL, and that of the carcinoembryonic antigen (CEA) reached 4.98 ng/mL. The values of CA-242, CA-50, and CA-199 were slightly higher than normal ones. The patient subsequently underwent a transrectal needle biopsy aimed at the low signal lesion of the prostate. The histopathological examination found no definite malignancy (**Figure 3A**).

**Figure 4:**
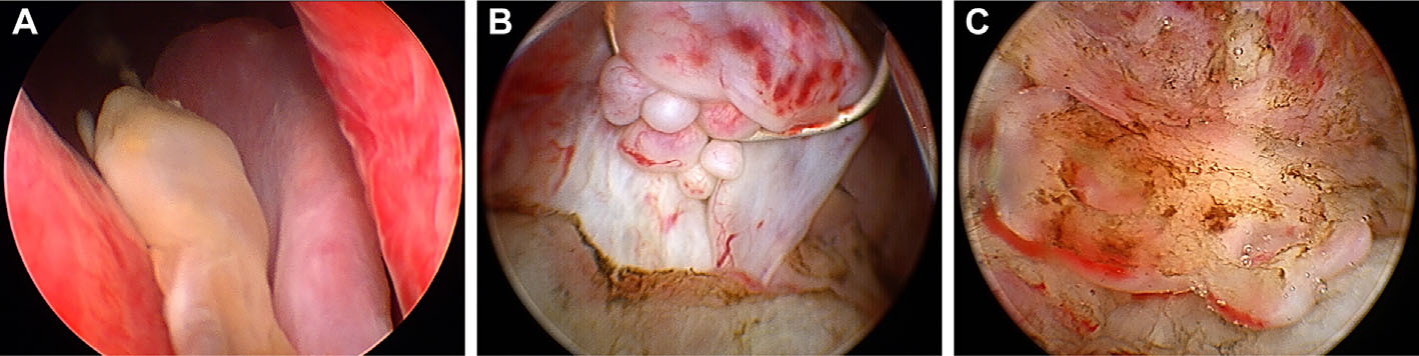
Urethrocystoscopic performance of the tumor. **(A)** Two cord-like neoplasm located at the apex of the prostate and extended from the right of verumontanum to the neck of the bladder. **(B)** A multi-chamber cystic mass surrounded a region of the prostate gland that lacked blood supply. **(C)** Mucous substance can be observed on the cut surface.

Three weeks later, this patient was hospitalized with dysuresia and transurethral plasma kinetic resection of the prostate (TUPKP) was accordingly performed to relieve the symptoms and confirm the diagnosis. It was noteworthy that urethrocystoscopy examined two cord-like neoplasms in the prostatic urethra, extending to the neck of the bladder. Both of them had pedicles that were located at the prostatic apex on the right side of the verumontanum (**Figures 4A, B**). The cord-like neoplasm was first removed from the pedicle, and then the right lobe of the prostate was resected. This part of the prostate tissue was surrounded by a multi-chamber cystic mass. There were clear boundaries between the cysts and prostate tissue. In the process of the resection, it was found that the surrounding prostate tissue had a tough texture and no blood supply (**Figure 4C**). For the purpose of pathological diagnosis, the surgery aimed to remove the whole tumor with clean margins. Surprisingly, postoperative pathology indicated multifocal mucinous adenocarcinoma with a Gleason score (GS) of 4 + 3 = 7 (**Figure 3B**). Further immunohistochemical staining showed sections were tested positive for PSA and prosaposin (PSAP) (**Figures 3C, D**), and negative for caudal type homeobox 2 (CDX-2), cytokeratin-20 (CK20), alpha-methylacyl-CoA racemase (AMACR, P504S), cytokeratin-5/6 (CK5/6), cytokeratin-7 (CK7), high molecular weight cytokeratin 34βE12, and transformation-related protein 63 (P63), and Mucin-2 (MUC2) staining revealed ∼20% positivity (**Figure 3E**).

Radical prostatectomy was performed one month after it was confirmed that the bone scan and colonoscopies demonstrated no abnormality and a follow-up visit was made for the patient for three years to date. The latest examination showed the patient had no biochemical recurrence and all tumor markers remained at normal levels. The MRI indicated the signal of the anastomosis area was normal and no enlarged lymph node was detected in the pelvic cavity.

## Discussion

MCP, also known as colloid adenocarcinoma, is considered as one of the rarest morphological variants of prostate cancer (PCa; 6, 9, 10). Most of patients with MCP are sensitive to androgens (9–11). The most common site of metastases is the bone (usually osteoblasts), followed by lymph nodes and lungs (5, 6). Diagnostic criteria for MCP were established in 1979, and then extended in 2000 and 2008: 1) Only radical prostatectomy specimens can be used for diagnosis, and it requires the presence of at least 25% of the original tumor composed of glandular tissue with extra luminal mucin. 2) Primary non-prostatic mucinous carcinoma must be excluded. 3) The growth pattern of the tumor should not be papillary. 4) Gleason score grading should be based on the underlying architectural pattern. 5) The involvement of urothelial type prostatic adenocarcinoma must be minimal or only secondary (2, 12–14). Although the original tumor should be composed of at least 25% glands with extra-luminal mucin to confirm the diagnosis, the clinical significance of this cut-off point is unclear (15). Furthermore, the volume and proportion of the mucinous component have no impact on prognosis (5, 7). Herein, the MCP and PCMF will be touched upon.

Significant changes have taken place in the criteria for grading mucinous adenocarcinoma (9, 12, 16, 17). Many pathologists were inclined to assign GS = 8 to all prostate mucinous adenocarcinoma (14). Nevertheless, on the 2014 International Society of Urological Pathology Consensus Conference reached a consensus, stating that the underlying structure of a tumor should serve as the basis for determining GS (18). Even so, it is a must for us understand that the hypothetical prognostic significance of grading derived in this way has insufficient evidence. The relationship between GS and the prognosis of mucinous adenocarcinoma has not been comprehensively elucidated (14). The GS assigned for mucinous adenocarcinoma is usually high, while its prognosis seems to be analogous to non-mucinous adenocarcinoma with the same GS. The average 5-year biochemical recurrence-free survival for patients with MCP was reported to be 87.5-100% (4, 7, 14).

The morphology of the mucus components is usually variable and has multiple forms in most cases. Common forms of the glands consist of cribriform, poorly formed, unitary well-formed, and fused one, whereas isolated cells, strings of cells, papilliform structures, and solid bunches are observed less often (7, 16). The immunohistochemical presentation of prostatic mucinous adenocarcinoma is similar to that of regular acinar prostate adenocarcinoma, often tested positive for PSA and prostatic acid phosphatase (PAP) (19). Only a minority of cases are negative for PSA and PAP, yet positive for CEA (2, 5). Most patients with prostate mucinous adenocarcinoma have the improved serum tPSA, with an average level of 9.0 ng/mL (14). Another study evaluated 143 samples with a mucinous component of 5–100% and found an average preoperative tPSA value of 7.8 ng/mL (7).

MUC2, a known suppressor of breast, pancreas, and colon adenocarcinoma tumor, was also detected in all MCP patients (20–22). Nevertheless, it remains unknown whether it will play a role in the behavior where the cancer seems relatively indolent. Similar to non-mucinous PCa, studies have found that the ETS-related gene (ERG) is tested positive in approximately half of MCP and PCMF patients (23, 24). While TMPRSS2-ERG fusion was identified in 83% of mucinous adenocarcinomas, its prognostic value has aroused controversy (25, 26). Some suggest that the fusion of these genes is associated with a worse prognosis (27, 28), while others have found a correlation between the fusion status and tumor stage, and it is not linked with recurrence or mortality (29, 30). Some studies have even indicated that there is no correlation with the tumor stage, GS, or biochemical recurrence-free survival (24, 31). Considering the prognosis of mucinous PCa, these studies may further confirm that TMPRSS2-ERG fusion fails to predict the prognosis of PCa.

The conventional interpretation method of MRI for non-mucinous PCa may fail to be applied to mucinous adenocarcinoma (32, 33). Typically, on T2-weighted (T2WI) MRI, almost all types of mucinous carcinomas in other organs display a high signal intensity and are therefore confused with necrotic tumors, effusions, and cysts (34). A study on four cases of mucinous adenocarcinoma found that all lesions appeared highly intense on T2WI MRI. This situation was especially so when the tumor was confined to the peripheral zone (PZ) where it was difficult to identify, under the circumtance of being isointense with the surrounding normal PZ tissue (35). A previous study manifested that mucinous prostate adenocarcinoma metastasis, which could not be detected by 18F-sodium-fluoride (Na-F) positron emission tomography/computed tomography (PET/CT) or 18F-fluciclovine PET/CT, could be identified by 68Ga-PSMA-11 PET/CT successfully, which might be utilized for differential diagnosis in the future (36).

MCPU is another variant of primary mucinous prostate gland tumor, arising from the prostatic urethra and commonly progressing rapidly (37). The tPSA value of these patients has never increased. Tumors are generally positive for CEA, CK7, and CK20 and negative for PSA and PSAP (38). It is worthwhile noting that mucinous carcinoma with signet-ring cells and signet-ring cell carcinoma also have mucinous features, making it particularly essential to distinguish these from mucinous adenocarcinoma, since these tumors are extremely aggressive, with no response to endocrine therapy, and there is zero rate of survival for 5-year patients (11, 39).

In this report, the tPSA level of the patient remained normal and the biopsy result revealed no definite malignancy. Non-mucinous PCa are often represented by hypointensity on T2WI MRI, whereas this lesion showed high signal in the periphery and low signal internally on T2WI MRI, which has greatly puzzled the authors. Accordingly, the low-signal shadow was targeted for needle biopsy and no malignancy was detected. For further diagnosis, TUPKP was subsequently performed and it could be observed under urethrocystoscopy that the surrounding mucus-rich tissue had a clear boundary with the internal one. Actually, mucinous carcinomas usually demonstrate hyperintensity on T2WI MRI. Coupled with the urethrocystoscopic manifestation and the pathological features, it was acknowledged that the periphery of the lesion was mucinous adenocarcinoma, while the low-signal internal tissue on T2WI MRI was prostate tissue lacking blood supply. This peculiar growth pattern of cancer has never been reported before. The lesion’s periphery was thin and contained much mucus, thereby making it difficult to get a specimen through puncture.

For lesions with highly suspected malignancy but negative results of needle biopsy, it is believed that transurethral resection specimen pathological examination can be employed for diagnosis, if the tumor is located in the central zone or transitional zone of the prostate. Prostatic mucinous adenocarcinoma seems to differ in the origin, growth pattern, and biological behavior from non-mucinous adenocarcinoma. Given the difficulty in diagnosing prostate mucinous adenocarcinoma, we hope this report could be conducive to clinicians, radiologists, and pathologists’ further understanding of this disease.

## Data Availability Statement

The datasets generated for this study are available on request to the corresponding author.

## Ethics Statement

Written informed consent was obtained from the individual(s) for the publication of any potentially identifiable images or data included in this article.

## Author Contributions

YZ, HS, JL, and HY collected and analyzed the patient’s clinical data and designed the research. ZY, KL, and HW performed the review of literature and drafted the manuscript. HS, WW, and ZW supervised the report and the publication process. All authors contributed to the article and approved the submitted version.

## Funding

This study was supported by Nanjing Medical Science and Technique Development Foundation (QRX17099) and National Natural Science Foundation of China (no. 81771640).

## Conflict of Interest

The authors declare that the research was conducted in the absence of any commercial or financial relationships that could be construed as a potential conflict of interest.
